# Neuromagnetic effects of pico-Tesla stimulation

**DOI:** 10.1088/0967-3334/36/9/1901

**Published:** 2015-08-06

**Authors:** Luzia Troebinger, Photios Anninos, Gareth Barnes

**Affiliations:** 1 Wellcome Trust Centre for Neuroimaging, Institute of Neurology, UCL, UK; 2 Laboratory of Medical Physics, Medical School, Democritus University of Thrace, Alexandroupolis 68100, Greece; g.barnes@ucl.ac.uk

**Keywords:** MEG, magnetic stimulation, pico-Tesla, theta rhythm

## Abstract

We used a double-blind experimental design to look for an effect of pico-Tesla magnetic stimulation in healthy subjects. Pico-Tesla stimulation is thought to increase the dominant frequency of 2–7 Hz oscillations in the human brain. We used magnetoencephalography to measure resting state brain activity. Each subject had two separate recording sessions consisting of three runs in between which they were given real or sham pT stimulation. We then tried to predict the real and sham stimulation sessions based on changes in the mean peak frequency in the 2–7 Hz band. Our predictions for these individual runs were 8 out of 14 at chance level (*p* = 0.39). After unblinding, we found no significant effect (*p* = 0.11) of an increase in the frequency range (2–7 Hz) across the subject group. Finally, we performed a Bayesian model comparison between the effect size predicted from previous clinical studies and a null model. Even though this study had a sensitivity advantage of at least one order of magnitude over previous work, we found the null model to be significantly (2000 times) more likely.

## Introduction

A number of studies have now suggested that pico-Tesla (pT) (where 1 pT = 10^−12^ T) range stimulation has some quantifiable benefit (Anninos *et al* 1991). Specifically, the electronic device invented by (Anninos and Tsagas 1995) is thought to increase the frequencies of endogenous brain activity of the (2–7 Hz) range towards frequencies of less than or equal to those frequencies of the alpha frequency range (8–13 Hz) of each individual subject (Anninos *et al* 2008). One possible electrophysiological explanation for the efficacy of pico-Tesla-magnetic-stimulation (pTMS) has been provided by the proposed ‘neural net model’ (Anninos *et al* 1989) which suggests that magnetic stimulation causes a temporally modulated neuronal inhibition in regions exhibiting abnormal activity in the frequency range of 2–7 Hz. This hypothesis is in concordance with data presented by other investigators (John 1967, Kaczmarek and Adey 1974, Ossenkopp and Cain 1988). Clinically, this technique is now regularly used in the Government General University Hospital, Laboratory of Medical Physics in the School of Medicine of DUTH University in Greece (Anninos *et al* 1991, 2000, 2006, 2007, 2008). All patients treated in the above references were referred from neurologists from other hospitals or neurology clinics in Greece. One potential reason for this clinical benefit is that it counteracts the rhythmic slowing of activity associated with conditions such as Parkinson’s disease (Stoffers *et al* 2007, Olde-Dubbelink *et al* 2013). In this study we set out to show the effect of pT stimulation in healthy subjects using state of the art magnetoencephalographic (MEG) recording protocols and a double blind experimental design.

In this paper we sought to objectively quantify the effect of pT stimulation based on the measurement of changes in the resting state rhythmic activity in healthy participants. One problem of longitudinal measurements with MEG is that the location of the MEG sensors with respect to the head is not fixed. Therefore one can expect a large amount of sensor level noise simply due to the change in head position between scanning runs. We recently demonstrated the use of custom made headcasts for MEG (Troebinger *et al* 2014). These casts fit the subject’s head internally and the MEG dewar externally and allow us to reposition the subject’s head to within around 1 mm on separate days. This attenuation of co-registration noise increases the channel level signal to noise ratio by a factor of 5 equating to a 25 fold reduction in scanning time required to see the same longitudinal sensor level effect (Troebinger *et al* 2014).

In this study we combine high sensitivity MEG recordings using a head-cast with pT stimulation of the cortex. The aim is to use very sensitive methods to identify any change in brain state consistent with our predictions that the pT helmet should increase the peak frequency within the 2–7 Hz band towards frequencies of less or equal to those frequencies of the alpha frequency range (8–13 Hz) for each individual subject (Anninos *et al* 1991, 2000, 2006, 2007, 2008).

## Methods

### MEG scanner

**Figure 1.**
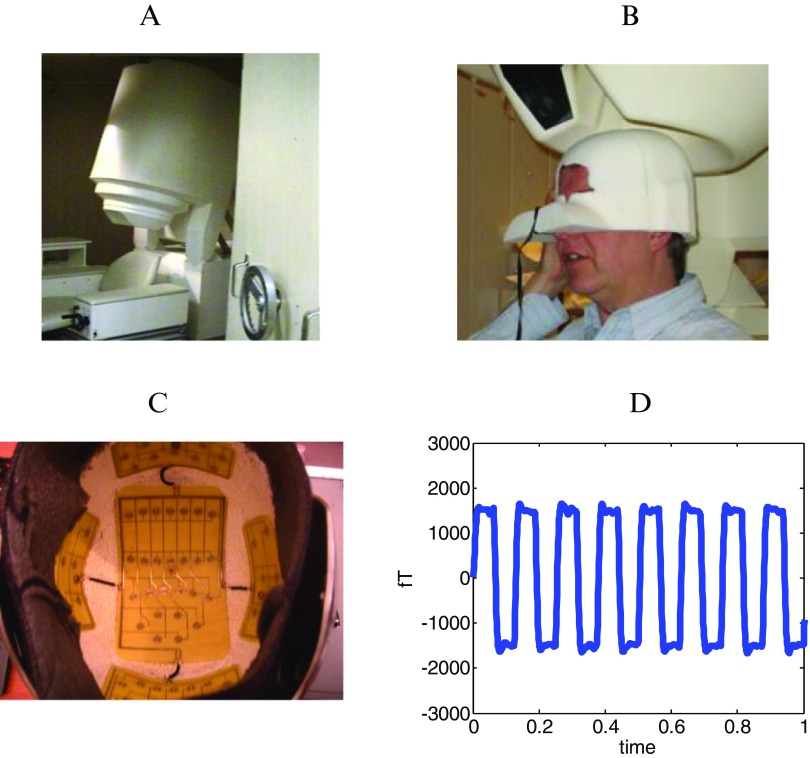
Panel A shows a 275 channel CTF Omega MEG system. Panel (B) shows the subject specific head-cast. The head-cast fits the subject internally and the MEG system externally. Panel (C) shows the configuration of the stimulation coils within the pT helmet device. The view here is from below showing the five coil groups: vertex, left temporal, right temporal, occipital and frontal coils (at top of picture). Panel (D) shows a schematic of the pT square-wave stimulation waveform as a function of time.

We used a 275 channel CTF Omega MEG system (figure 1(A)). Sampling rate was set at 600 Hz with anti-aliasing filters at 150 Hz. All seven subjects were healthy male volunteers of age range 20–55 years. All procedures were carried out in accordance with UCL ethics.

### Experimental protocol

Prior to MEG scanning each subject undertook a structural MRI (see below) in order to create a custom-made polyurethane head-cast (figure 1(B)). Participants were scanned in three separate sessions typically separated by 1 week. Each subject wore their individual head-cast during the MEG scans and was therefore precisely repositioned at the same location (over visits) with respect to the MEG sensors. The subject had no task and was asked to sit comfortably.

### Calibration session

The first session (session 0) consisted of a 2 min resting state MEG scan. These data were subsequently used to establish the subject’s alpha frequency in the range of (8–13 Hz), for calibration of the pTMS electronic device.

### Testing sessions

In the second (session 1) and third (session 2) scanning sessions, the protocol was as follows. At all times the pT helmet was set to real or sham stimulation by a third party. Neither the researcher nor the participant were aware of the state of the device.

First, two minutes of pre-stimulus baseline MEG data were recorded (run A). Next, 2 min of real or sham pTMS stimulation were administered with the subject sitting comfortably just outside the scanner room. Following these 2 min of stimulation, a further 2 min of resting state MEG data were acquired (run B). This was followed by another 2 min of stimulation- in this case the device was switched from sham to real or vice versa (by the third party) and two more minutes of MEG scanning were carried out (run C).

The third session followed exactly the same protocol as the second, with the only difference being that the order of real versus sham stimulation was reversed (i.e. if the order was A-Stim-B-Sham-C for session 1, it was A-Sham-B-Stim-C for session 2).

### The pTMS electronic device

The pTMS electronic device is a modified helmet containing up to 122 coils which are arranged in five array groups, so as to cover the main five brain regions (frontal, vertex, right and left temporal and occipital regions) of the subject (figure 1(C)). It is designed to create pT range modulations of magnetic flux in the alpha frequency range (8–13 Hz) of each healthy human subject. The pTMS device was configured for each individual to generate a square wave (so as to resemble the firing activity of neurons in the brain) (Anninos *et al* 1970) modulated magnetic field at the individual’s mean peak alpha frequency—generated in the subject’s occipital lobe (Anninos and Tsagas 1995). A schematic of the alpha wave generated by the electronic device can be seen in figure 1(D) and more details, including the corresponding power spectrum, can be found in Anninos *et al* (1991).

We added an extra hidden switch to the pTMS device to disable current flow to the coils. This switch, controlling real or sham stimulation, was operated by a member of the technical support team, so that neither the subject nor the experimenter were aware of whether sham or real stimulation was applied (double blind design).

### Spectral estimates

In both the calibration and subsequent analysis stages we computed mean peak frequency for specific channel groups. We calculated the root mean square (rms) amplitude spectrum in each 2 min recording session using 70 non-overlapping data segments of 1024 samples. The mean level of each of these segments was removed and the data multiplied by a Hanning window. We computed the absolute value of fast-Fourier transform of each of these windows and averaged these to create a single rms amplitude spectral estimate per MEG channel. We then looked within the band of interest (alpha for calibration, 2–7 Hz for analysis) and looked at the frequency corresponding to peak power in this band. We did this for each channel within the group (eg occipital for calibration) in order to get a single mean peak frequency per channel group. Channels were grouped according to the MEG manufacturer’s (CTF’s) canonical labelling scheme (RT right temporal, LO left occipital, etc); there were 10 such channel groups, each containing between 18 and 33 channels.

### MRI acquisition

MRI data was acquired using a Siemens Tim Trio 3T system (Erlangen, Germany). The subject lay in the supine position. The body-transmit coil was located inside the bore of the scanner for detection of the MRI signal. The MRI data was acquired using a 3D FLASH sequence for optimal scanning efficiency (Frahm *et al* 1986). The following acquisition parameters were used: field of view: (256, 256, 208) mm along the (phase (A-P), read (H-F), partition (R-L)) directions, image resolution: 1 mm^3^. The repetition time TR was set to 23.7 ms and the excitation flip angle was set to 20° to yield good T1-weighted contrast, standard in most anatomical applications (Helms *et al* 2008). 8 echoes were acquired following each excitation and averaged offline to produce one anatomical image with optimal signal-to-noise. A high readout bandwidth was used to preserve brain morphology and no significant geometric distortions are expected in the images. Padding was used to minimize subject motion but some residual effects might remain present in the MRI images. The total acquisition time was 21 min 07 s.

### Analysis

We performed our analysis in two stages. Firstly we (PA and GB) tried to blindly identify real from sham runs based on the predicted frequency increase due to pT stimulation.

### Prediction of sham and stimulus runs

In order to increase our statistical power we treated each visit (from sessions 1 and 2) independently giving 14 visits. In each of these visits, there are three data sets (A, B, C) and the task is to decide where the sham stimulation was delivered (before recording B or before recording C).

We first computed the mean peak frequency (see above) in the 2–7 Hz frequency band over spatially distinct MEG channel groups and over all these groups combined for each of the three runs, A, B, C. This gave a mean peak theta frequency within channel group i in runs A, B, C of $\overline{{{f}_{\text{A},i}}}$, $\overline{{{f}_{B,i}}}$, $\overline{{{f}_{C,i}}}$ respectively.

We then calculated the increase in frequency ($\Delta f$) from sham to real stimulation under two possible scenarios. Firstly, assuming run C was the sham: therefore we take the mean peak frequency from run B (the post-stimulation run) and subtract the average mean peak frequencies of runs A and C (the baseline and post-sham runs) for all channels in each brain region for a specified visit.

1 \begin{eqnarray*}\Delta {{f}_{\text{Csham},i}}=\overline{{{f}_{B,i}}}-\frac{\overline{{{f}_{A,i}}}+\overline{{{f}_{C,i}}}}{2}\end{eqnarray*}

Or assuming run B was the sham

2 \begin{eqnarray*}\Delta {{f}_{\text{Bsham},i}}=\overline{{{f}_{C,i}}}-\frac{\overline{{{f}_{A,i}}}+\overline{{{f}_{B,i}}}}{2}\end{eqnarray*}

Table 1 shows these frequency differences within different channel groups for a single subject visit. Based on the mean frequency difference across all channel groups (bottom of table 1) we were able to make a prediction of the likely stage (B sham or C sham) of pT stimulation in each of the 14 recording sessions.

**Table 1.** Shows exemplar data from one subject visit. Each row summarizes a group of MEG sensors (LT—left temporal, LC—left central, LF—left frontal, LP—left parietal, LO—left occipital, RT—right temporal, etc). The second column shows the change in mean peak frequency in the 2–7 Hz band between the average peak frequency in runs A and B as compared to C (this corresponds to the assumption ‘B sham’); and the third column shows the average peak frequency in runs A and C as compared to run B or ‘C sham’. For example, looking at MRP we see that the mean peak frequency over these channels has increased when we assume that run C is the sham. The last row shows the average frequency change over all channel groups under the two hypotheses. In this case therefore, as we assumed that the pT device would give rise to a frequency increase, we would predict that C was the sham condition.

Chan group	Assume B sham frequency change (Hz)	Assume C sham frequency change (Hz)
MLF	−0.5	1.4
MRF	−0.4	0.2
MLT	−0.3	0.5
MLC	−1.2	1.7
MRC	−1.4	2.1
MRT	−0.4	−0.1
MLP	−1.7	2.2
MRP	−2.5	3.0
MLO	−0.3	−0.4
MRO	−2.9	0.1
Mean all groups	−1.16	1.07

Specifically, when Δ *f* _Bsham_  >Δ   *f* _Csham_ we predicted B to be the post-sham recording and C to be the post-stimulation run; and for Δ *f* _Csham_  >  Δ *f* _Bsham_ we predicted C to be the post-sham and B to be the post-stimulation run for (where Δ *f* _Csham_ and Δ *f* _Bsham_ are mean peak frequency change over all channel groups).

## Results

### Visit-level scoring of the predictions

During each visit we attempted to determine the order of stimulation (B sham or C sham) based on the mean peak frequency change over channel groups as shown in table 1. On each of the visits we based our prediction (B sham or C sham) on whichever order gave rise to the largest positive change in mean peak frequency.

**Figure 2.**
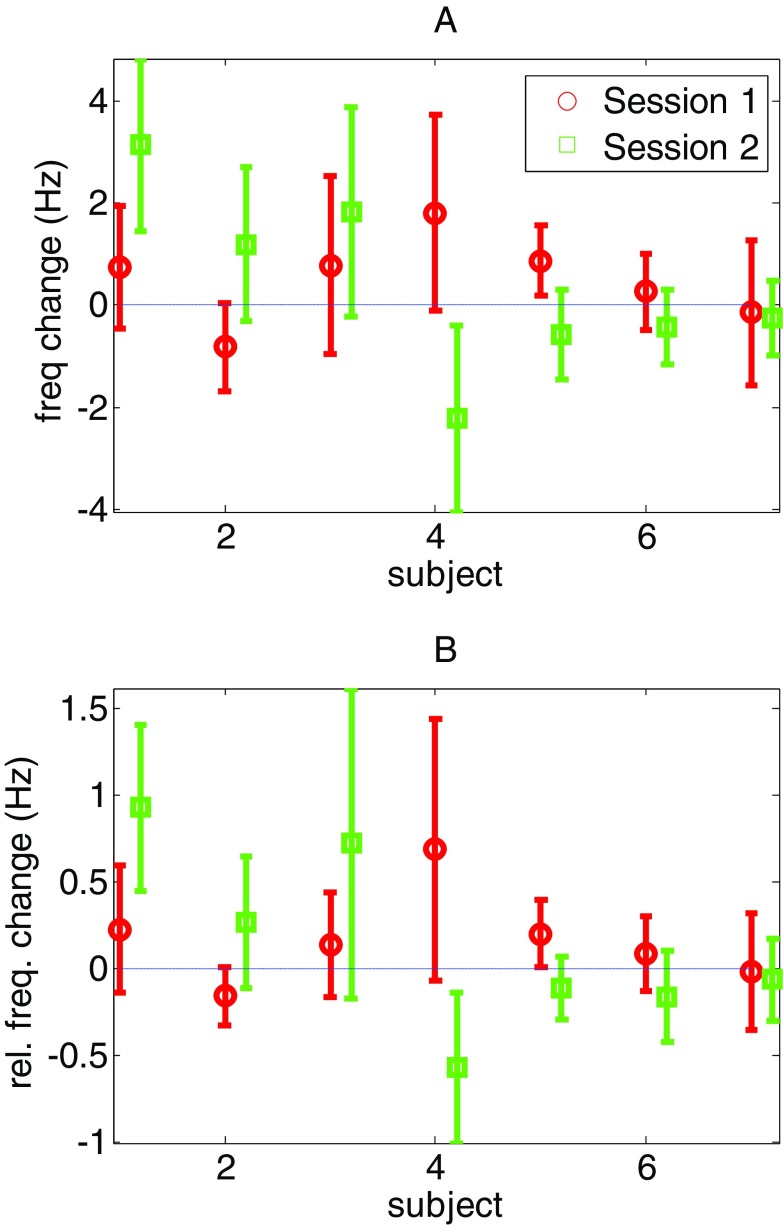
The mean peak frequency differences for each of the two sessions in seven different subjects (comprising the 14 visits). The error bars show standard deviation over channel groups. Note that in eight of the 14 visits the mean peak frequency difference increased; and hence our predictions for the effect of the pT stimulation were correct 57% of the time. Panel B shows the same data but in terms of relative frequency change (with respect to mean session frequency).

In figure 2, based on knowledge of the true stimulation sequence, we can show the true per-visit effect of pT stimulation. Mean values above zero indicate that our prediction for this particular visit was correct (in 8/14 cases). Based on the binomial test, the probability for correctly selecting eight or more events, each with a probability of 0.5, from 14 by chance is *p* =  <0.39. That is, at a per-visit level we see no significant effect of pT stimulation (*p*  <  0.39).

### Group level frequency change due to stimulation

The pT helmet literature suggests that the post-stimulus runs should have a higher frequency than the post-sham runs. Although this was not significantly predictive of stimulation at a single subject level (*p*  <  0.39), we now test whether or not the average frequency across all subject visits might have increased. In order to do this we can compare the observed average change in frequency (over all subjects and sessions) against the average frequency change when stimulus labels are randomly assigned to visits (real or sham first). We used 1000 random permutations and found that the observed mean frequency difference (0.42 Hz) was not significant (*p* = 0.11).

It is possible that the group effects were diluted by a variation in theta frequency over subjects. We therefore made the same test again by looking at the relative peak theta frequency change. We calculated this in two ways: as the frequency change (due to stimulation) relative to run A (baseline) and as the frequency change (due to stimulation) relative to the mean session frequency (average of runs A, B and C). We performed this second measure as the correlation between peak frequency in successive run As on each subject was relatively poor (*n* = 7, *r* = 0.46, *p* = 0.28) as compared to the mean frequency over A, B and C which was more robust (*n* = 7, *r* = 0.84, *p*  <  0.0167). For both relative measures, using the same permutation test above, we found no significant effect of stimulation (*p* = 0.08, 0.09 respectively).

In the above analysis we treated the two visits of each subject as independent (*n* = 14). When averaging the two visits from each subject (*n* = 7) we performed the same permutation test and also found also found no effect on the average absolute or relative (to mean session) frequency change of *p* = 0.13 and *p* = 0.1 respectively.

### Between session effects

It is possible that although no mean change is observed, for a given subject, some MEG channel groups are consistently affected by pT stimulation. In order to examine this we took the channel group with the greatest change in frequency in session 1 and tested how likely it would be to show a change (in the correct direction) in session 2 for the same subject. If there were a consistent effect of pT stimulation within a subject, then all changes in the second session should be positive. However here we find no significant difference with a trend for a decrease in frequency in this pre-selected channel group (*t* =  −0.511, *df* = 6, *p*  <  0.627).

### Residual effects

In rat models (Welker *et al* 1983) the effects of externally applied magnetic fields have been shown to persist for up to two hours. We therefore did want to rule out the (admittedly unlikely) possibility that the lack of consistency within subjects could be due to some residual effects of stimulation in session 1 during recording of session 2 (two weeks later). For example, session 1 might have increased a subject’s mean peak theta frequency and this might not have returned to pre-stimulation level by the time session 2 began. In order to test for this, we compared the mean peak frequency between run A of session 1 and run A of session 2. Run A is a baseline scan before any stimulation and so a difference between session 1 and session 2 would indicate that some residual effect of stimulation had contaminated session 2. We found no change in frequency over sessions with a trend towards a frequency decrease (*t* =  −0.5929, *df* = 6, *p* = 0.57).

### Topography of the frequency change due to stimulation

**Figure 3.**
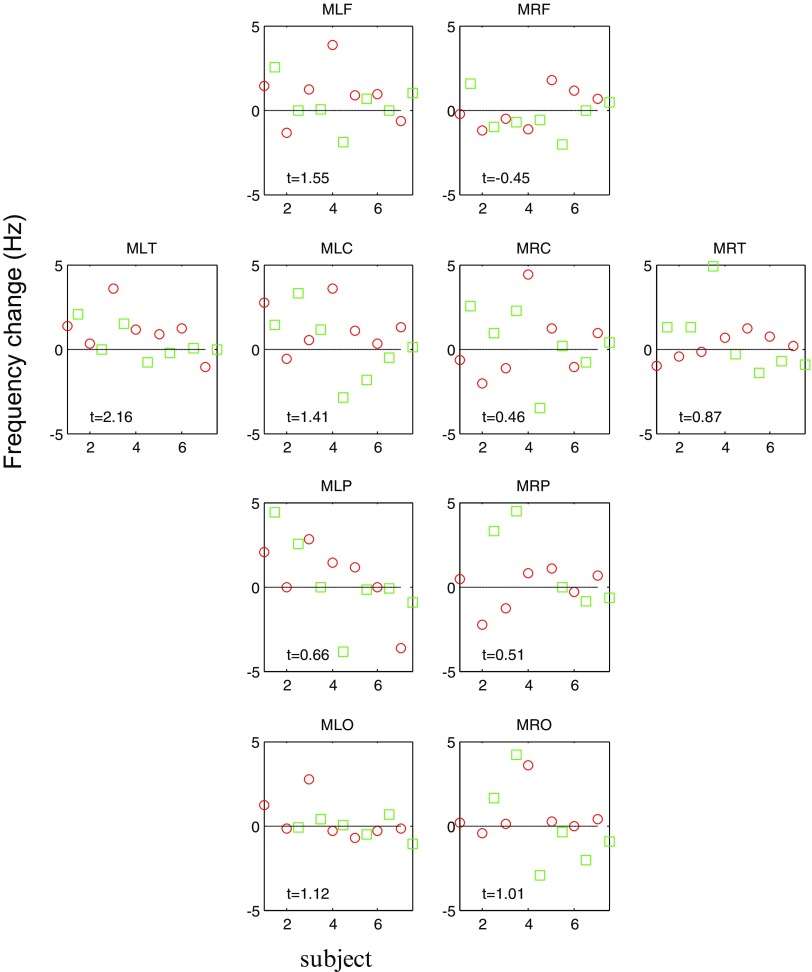
Change in frequency across cortical regions. This plot is essentially an expanded version of figure 2, detailing changes in frequency over different channel groups. Mean frequency change in Hz (*y* axis) for the two sessions (session 1 red circles, session 2 green squares) over seven subjects (*x* axis); positive values indicate an increase in frequency between sham and stimulation conditions. Also shown are one sample t statistics based on the 14 values for each channel group. Note that the most consistent effect of stimulation was observed in the left temporal lobe (MLT, *t* = +2.16) and that all *t*-statistics are positive except for the right frontal channel group (MRF).

Although we found no global effects of stimulation, it is possible that some cortical regions are more susceptible to pT stimulation than others. In figure 3 we re-plot figure 2, but broken down into different cortical regions. Also shown are t-statistic values looking at the 14 values (two sessions, seven subjects) for that particular channel group. The left temporal (MLT) channel group showed the maximal effect of pT stimulation (*t* = 2.16). One point of interest is that the channel group level changes, across all subjects and both sessions, are generally positive (*t*  >  0 in all groups except the right frontal channels MRF).

### Topography at single channel level

**Figure 4.**
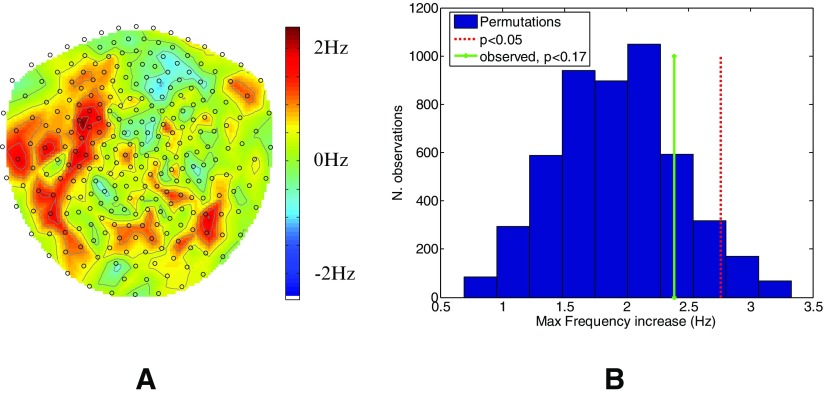
Change in frequency across channels. Panel (A) shows the mean theta frequency difference (colour scale) between stimulation and sham conditions in each channel across all subjects. The channels to the front of the head are towards the top of the page. The histogram in panel (B) shows the null distribution of this frequency difference (essentially randomly relabeling sham and stim conditions). The red dotted line shows the 95 percentile of this null distribution and the solid green line shows the largest change in frequency actually observed (*p*  <  0.17).

In order to verify that a channel level effect had not been diluted within channel groups we looked across visits for a significant change in the 2–7 Hz frequency band at any single channel over all subjects. We asked if there were any channels that showed a peak frequency change (in the 2–7 Hz band) between stimulation and sham of more than one would expect by chance. Figure 4(A) shows the topography of the average peak frequency change due to pT stimulation in the group. In order to assess whether these changes could have arisen by chance, we randomly permuted the sign for each subject 5000 times to produce a null distribution. Figure 4(B) shows this null distribution (blue histogram) alongside the *p*  <  0.05 threshold for this distribution (red dotted) and also the maximum observed frequency increase in the range (2–7 Hz) in panel A (green solid). Again it is clear that there is no significant (*p*  <  0.17) change in the frequency range (2–7 Hz) with stimulation.

### Bayesian perspective

Using the classical frequentist statistical approach above we have not been able to reject the null (that the pT stimulation has no effect). The frequentist approach also precludes us from accepting the null. In order to produce a conclusive statement about the effect of pT stimulation in healthy controls we make use of a Bayesian perspective, which allows us to ask which of two models (one model assuming the previously observed clinical effect size and one null model) is most likely given the data we observed.

**Figure 5.**
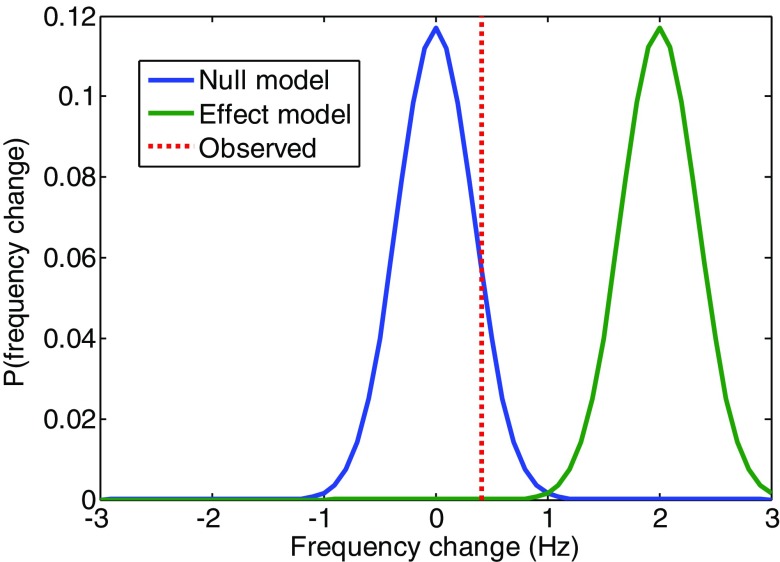
Bayesian model comparison. The probability distributions for the null (0 Hz) and effect (2 Hz) models (as blue and green solid curves respectively) of frequency change alongside the observed mean frequency change due to stimulation (red dotted). At the observed frequency change (0.415 Hz) the ratio of the probability of the null to the effect model (where they cross the dotted line) is 2200 or a log Bayes factor of 10.04.

We took the clinical model to be a 2 Hz frequency increase based on previous work showing a (2–7 Hz) frequency change of greater than 2 and less than 6 Hz (Anninos *et al* 1991, 2000, 2006, 2007, 2008, O’Clock 2003). We compared this to a null model in which the frequency change was 0 Hz. The two competing models were therefore two Gaussians with means of 0 and 2 Hz (for null and effect models, respectively) with dispersion equal to the observed standard error on theta frequency change over visits. These Gaussians are plotted in figure 5 alongside the observed mean frequency change (dotted line) over subjects. By reading off the probability of each of the models at the observed frequency change we can calculate their relative probability or Bayes factor. We find the null model to be 2200 times (or log Bayes factor = 10) more likely than the effect model.

## Discussion

In this study we set out to replicate the effects of increased cortical endogenous frequencies in the 2–7 Hz band due to the effect of pT stimulation (Anninos *et al* 1991, 2000, 2006, 2007, 2008) in a group of healthy controls. We used a state-of-the-art head-cast design to ensure that the MEG measurements were of the highest possible precision.

The experimental design was double-blind (PA remains blinded to the true scanning order) and we first made predictions of the true order of stimulation based on the mean peak theta frequencies observed in the data. After unblinding we found that we had correctly predicted the order of stimulation in eight of the 14 visits (57% correct). This performance is in line with what one would expect by chance (*p* = 0.39), that is one would perform equally well or better by guessing around 40% of the time. We then went on to look for more subtle effects which might not be manifest at a single subject level but show up in the group. Here we found that the average frequency change we observed between sham and stimulation runs remained non-significant (*p*  <  0.11).

We then looked at whether the effect of pT stimulation was consistent across participants. We found that the channel group showing maximal frequency change in the first session was unlikely to show a similar positive effect during the second session (*p*  <  0.627). We tested whether the session 2 scans could have been compromised by a residual effect of pT stimulation two weeks after session 1. We found no suggestion (*p*  <  0.57) that this could be the case.

We should note that by treating the two visits as independent in the group data we are assuming the same effect in all subjects (i.e. a fixed effects analysis) and that therefore the limiting factor is measurement noise (rather than inter-subject variability). We note that the over-estimation of the degrees of freedom should tend to bias our findings in an anti-conservative direction, yet we still see no effect. We also found no effect when averaging the data from the two visits of each subject.

Importantly, this study has at least an order of magnitude more sensitivity than any previous work. This is due to two main factors- the use of the headcast reduces the MEG sensor noise due to misalignment of head locations during the two visits by a factor of 5 (Troebinger *et al* 2014). Secondly, previous work has shown effects in single subjects (*n* = 1) whereas here we used a cohort of 7 participants each recorded over two visits. Finally, in order to test whether the previously observed frequency increase could potentially underlie these data, we compared this to a null model of 0 Hz frequency change and found the null model to be 2200 times more likely to have generated these data.

We therefore have to question why our results do not accord with the clinical findings. One possible reason is that here we used healthy controls and not patients. Another reason is that this is the first ever double blind study to assess the impact of the pT helmet. It will therefore be important that future clinical studies consider this and make use of double-blind paradigms so as to rule out the possibility that the benefits observed are due a placebo effect.
